# The Molecular Mechanism of Traditional Chinese Medicine Prescription: Gu-tong Formula in Relieving Osteolytic Bone Destruction

**DOI:** 10.1155/2022/4931368

**Published:** 2022-07-13

**Authors:** Jinyuan Chang, Zhenglong Jiang, Wei Jin, Yaohan Wang, Jie Li, Jiayang Chen, Hao Li, Li Feng

**Affiliations:** ^1^National Cancer Center/National Clinical Research Center for Cancer/Cancer Hospital, Chinese Academy of Medical Sciences and Peking Union Medical College, Beijing 100021, China; ^2^Department of Oncology, Beijing Hospital of Integrated Traditional Chinese and Western Medicine, Beijing 100039, China; ^3^Beijing University of Chinese Medicine, Beijing 100029, China

## Abstract

Bone metastasis is a common complication in patients with advanced tumors, causing pain and bone destruction and affecting their quality of life. Typically, complementary and alternative medicine (CAM), with unique theoretical guidance, has played key roles in the treatment of tumor-related diseases. Gu-tong formula (GTF), as a representative prescription of traditional Chinese medicine, has been demonstrated to be an effective clinical medication for the relief of cancer pain. However, the molecular mechanism of GTF in the treatment of osteolytic metastasis is still unclear. Herein, we employ network pharmacology and molecular dynamics methods to uncover the potential treatment mechanism, indicating that GTF can reduce the levels of serum IL6 and TGFB1 and thus limit the scope of bone cortical damage. Among the active compounds, sesamin and deltoin can bind stably with IL6 and TGFB1, respectively, and have the potential to become anti-inflammatory and anticancer drugs. Although the reasons for the therapeutic effect of GTF are complex and comprehensive, this work provides biological plausibility in the treatment of osteolytic metastases, which has a guiding significance for the treatment of cancer pain with CAM.

## 1. Introduction

Many advanced malignant tumors metastasize to the bones of the human body. Tumor cells that escape from the primary tumor are fixed in the niche of the bone microenvironment, which breaks the dynamic balance of normal bone remodeling, resulting in bone destruction and osteolytic metastasis. Osteolytic metastasis can cause a variety of skeletal-related events (SREs), such as severe pain, pathological fracture, and spinal cord compression, greatly affecting the quality of life of patients [1]. Although treatments for osteolytic metastasis have little effect, the incidence of SREs can be minimized by adopting bone-targeted agents, surgery, radiotherapy, and palliative treatment [2].

Interleukin-6 (IL6), as a key regulator of bone remodeling, is produced by tumor cells through autocrine and paracrine mechanisms in the bone microenvironment. It can combine with the IL6 receptor, promote the proliferation of osteoclasts, and play an important role in the occurrence of osteolytic bone destruction. Thus, drug research targeting IL6 has attracted great interest [3–5].

Traditional Chinese medicine, as a kind of complementary and alternative medicine (CAM), has unique advantages in the treatment of bone metastasis, especially in analgesia and bone protection, improving the quality of patient life and prolonging overall survival [6, 7], receiving many studies. According to pharmacological studies of Yishengukang decoction, Wenshen Zhuanggu formula, the Kampo formula, and Gu-tong formula (GTF, Patent No.: 201410415620.1), regulating the dynamic balance of osteoblasts and osteoclasts has been demonstrated to be the key of traditional Chinese medicine in the treatment of bone metastasis [8–11]. However, further research is hampered by complex components and diverse mechanisms [12].GTF, for instance, is prepared from a formula of nine Chinese medicines, including Flos Caryophylli (Ding Xiang, DX), Radix Aconiti Lateralis Preparata (Fu Zi, FZ), Rhizoma Zingiberis (Gan Jiang, GJ), Cortex Cinnamomi (Rou Gui, RG), Scorpio (Quan Xie, QX), Pseudobulbus Cremastraeseu Pleiones (Shan CiGu, SCG), Radix Clematidis (Wei Ling Xian, WLX), Rhizoma Curculiginis (Xian Mao, XM), and Herba Asari (Xi Xin, XX). Because of the mixing and doping of components, the research strategy of “one drug-one target” is not suitable for a comprehensive understanding of the prescription.

With the development of pharmacotherapy, it is crucial to comprehend the interaction between drugs and target molecules [13]. Various interactions, including hydrogen bonding and hydrophobic interactions, have been shown to be related to the maintenance of receptor conformation and the binding affinity between ligand and receptor [14–16]. The conformation simulation of ligand-receptor binding based on computational chemistry has been widely used in the study of pharmacodynamics and pharmacokinetics [17, 18]. The potential mechanism of docetaxel combined with anastrozole and the drug binding mode of cyanidin with the human serum albumin holo-transferrin complex were theoretically expounded [19, 20]. Simultaneously, the theoretical research was proven to be parallel to the experimental data, which fully demonstrated the reliability of molecular simulation technology in the field of drug research and development.

Herein, by adopting the methods of network pharmacology and molecular dynamics, we comprehensively analyzed the interaction networks between drugs and targets and clarified the binding modes of residues and compounds between proteins and ligands from the perspective of microscopic force, which provides a new strategy for the holistic, systemic, and comprehensive understanding of the mechanism of traditional Chinese medicine and developing active new drugs.

## 2. Methods

### 2.1. Screening of Active Compounds of GTF for Transdermal Absorption

The active compounds of GTF were screened by the TCMSP database, a unique systems pharmacology platform of Chinese herbal medicines, based on the conditions that the molecular weight was less than 500, the drug-likeness exceeded 0.18, and the logarithm of octanol/water partition was between 1 and 4 [21]. According to quantitative structure-activity relationship (QSAR) models and the drug molecular 3D similarity method, the potential targets of the active compounds with confidence and similarity thresholds greater than 90% and 1.2, respectively, were predicted by ChEMBL and ChemMapper databases [22–24].

### 2.2. Preparation of Potential Targets for Osteolytic Metastasis

The potential targets of the osteolytic metastases were obtained by combining the targets from GeneCards and OMIM databases with a correlation score > 3 [11].

### 2.3. Analysis of the Differentially Expressed Genes (DEGs) of Bone Metastasis in Lung Cancer Based on the GEO Database

The search parameters “bone metastases” and “Homo sapiens” (porgn: txid9606) were used to retrieve the publicly available lung cancer bone metastasis data set in the Gene Expression Omnibus (GEO) database. The criteria for the inclusion of the project and the sample are shown according to the following conditions: (1) the type of cancer is lung cancer, (2) sample size *n* > 10, (3) mRNA expression profile of cancer cell lines, (4) microarray data, (5) human lung adenocarcinoma cell line, and (6) the control cell line did not have a high bone metastasis ability. The GEOquery R package was used to obtain data sets. The following conditions were used to process the gene expression matrix: (1) probes with an expression value of 0 were removed; (2) probes without corresponding genes were removed; (3) multiple probes correspond to one gene, and the mean value was taken; and (4) carry out Limma (normalize between arrays) standardization [25].

The Limma R software package was used to analyze differentially expressed mRNAs between lung adenocarcinoma cell lines and bone metastatic lung adenocarcinoma cell lines at the threshold of logFoldChange (FC) > 1 or <- 1 and *P* value < 0.05 [26].

### 2.4. Construction of the Effective Compound-Target Network of GTF

The active compound-target network of GTF was established by Cytoscape 3.7.2 with the highlight of the related targets in the treatment of osteolytic metastases, followed by topological calculation of the degree value of each node to explore the key compounds in the formula.

The protein-protein interaction (PPI) network and related key genes (densely connected regions) were obtained by importing the GTF-disease intersection targets into the STRING 11.0 database and applying the “Molecular COmplex Detection” (MCODE) algorithm.

### 2.5. KEGG Pathway Enrichment Analysis

To further understand the function of active compounds, KEGG pathway enrichment analysis for intersection targets of GTF was conducted on clusterProfiler of RStudiov3.63 with an adjusted *P* value < 0.05 and the count of genes > 5 [27].

### 2.6. Chemical Space Analysis of Active Compounds in GTF

The chemical space of active compounds in GTF was analyzed by a total of 27 dimensions of the chemical and physical properties (found in the PubChem database) and the ASMET and toxicity properties (calculated by Discovery Studio, using QSAR and QSTR models, respectively). Principal component analysis (PCA) was conducted using Origin v2021 [28]. The mean IC50 (IG50) values of all the human cell lines were calculated according to the half maximal inhibitory concentration (IC50) and the half maximal inhibitory concentration on cell growth (GI50) collected through PubChem, DrugBank, ChEMBL, and related literature to explore the inhibitory effects [29].

### 2.7. Animal Studies

Twenty-four C57BL/6 mice aged six to eight weeks were randomly separated into the sham operation group (SOG), model group (MG), and Gu-tong formula group (GTF). Lewis cells in logarithmic growth phase were prepared into a 1 × 10^6^ cells/ml single-cell suspension with PBS. Five microliters of Lewis cells was injected into the left posterior tibia of MG and GTF mice, and normal saline was injected into SOG mice. All mice were sealed with bone wax to establish the animal model. The mice in the GTF group were given 25 g/kg GTF extract once a day for 21 days and were sacrificed at the end of the treatment period. Serum was collected to determine the concentrations of interleukin 6 (IL6) and transforming growth factor-beta 1 (TGFB1). The tumor-bearing tibia was preserved and fixed in 4% paraformaldehyde for bone mineral density (BMD) determination by contouring a region of bone destruction setting in the whole irradiated tibia [30].

Animal experiments were carried out in strict accordance with ARRIVE guidelines 2.0. This work was approved by the Animal Care Committee of National Cancer Center, National Clinical Research Center for Cancer, Cancer Hospital, Chinese Academy of Medical Sciences, and Peking Union Medical College (Beijing, China) (Permit Number: NCC2021A001).

### 2.8. Molecular Docking and Molecular Dynamics

Three-dimensional crystal structure coordinate files of human IL6 and TGFB1 were retrieved from the Protein Data Bank (PDB) (PDB ID: 1P9M and 3KFD, respectively), and potential binding sites were found by Discovery Studio based on the protein cavity. The structural formulas of key compounds were obtained from the PubChem database, and the force field of the compounds was optimized [31]. Molecular docking was performed using AutoDock software based on the Lamarck genetic algorithm [32]. Binding energy was used to screen for the most stable compound with proteins. The molecular dynamics study of the most stable binding compounds (IL6 and TGFB1) was carried out at 10 ns by applying position restraints and energy minimization and equilibrating at 300 K and 1 bar [33].

## 3. Result

### 3.1. The Prediction of Active Compounds and Potential Targets of GTF and Osteolytic Metastasis by Database Screening and DEG Analysis

Due to the unique physiological characteristics of the skin, the transdermal absorption of compounds requires multiple distribution processes in a hydrophilic/lipophilic environment. Therefore, the physical and chemical properties of compounds play key roles in the transdermal transport rate.

The compounds with transdermal absorption potential and their molecular formulas were screened and obtained from the TCMSP database and PubChem database, respectively. The potential targets were predicted by the TCMSP, ChEMBL, and ChemMapper databases. Note that GTF contains 9 kinds of traditional Chinese medicine with complex chemical components. Through database screening and prediction, 37 potential compounds (Supplementary Materials Table S1: the potential transdermal absorption compounds of GTF) and 282 potential therapeutic targets were obtained for further analysis.

**Figure 1:**
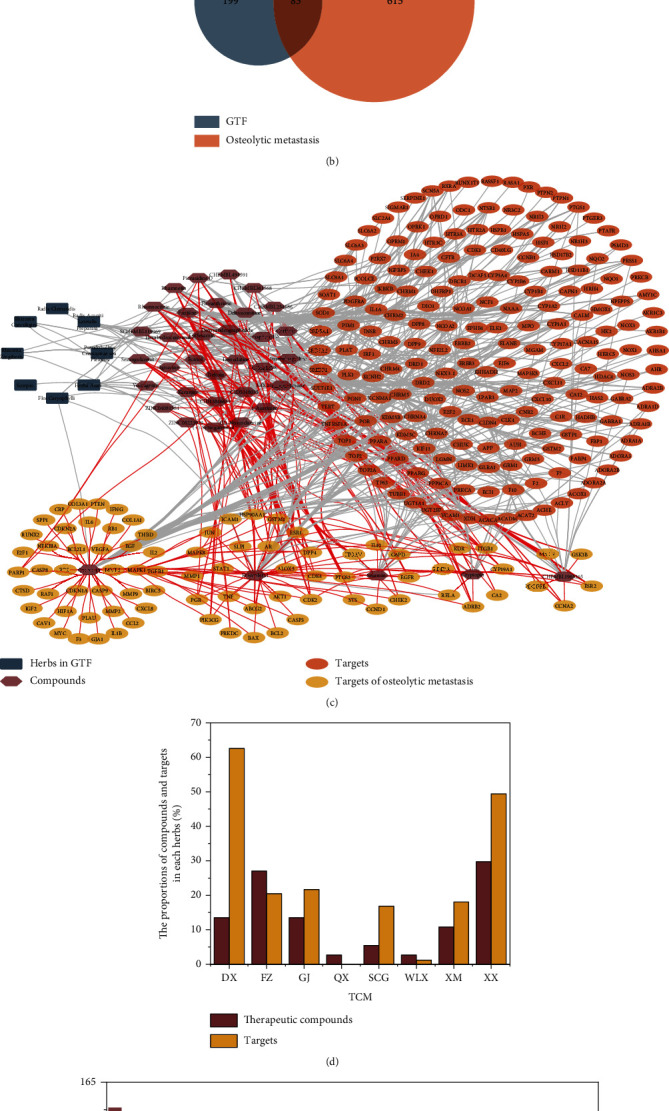
Network pharmacology analysis of GTF. (a) DGE analysis of bone metastasis of lung cancer based on the GEO database. (b) The number of targets for the treatment of osteolytic metastasis with GTF. (c) The interaction network of herb-compound-target. (d) The proportion of compounds and targets in each herb. DX: Flos Caryophylli (Ding Xiang); FZ: Radix Aconiti Lateralis Preparata (Fu Zi); GJ: Rhizoma Zingiberis (Gan Jiang); QX: Scorpio (Quan Xie); SCG: Pseudobulbus Cremastraeseu Pleiones (Shan CiGu); WLX: Radix Clematidis (Wei Ling Xian); XM: Rhizoma Curculiginis (Xian Mao); XX: Herba Asari (Xi Xin). (e) The key compounds in GTF.

The GEO database was used for the analysis of DEGs in bone metastasis of lung cancer. A total of 1919 entries were retrieved, of which the first 382 were independent series. According to the screening conditions, 3 available lung cancer bone metastasis queues were obtained, including GSE76194, GSE29391, and GSE10096, with 22 samples. As shown in Figure 1(a), 365 targets (out of 928 gene targets with a *P* value less than 0.05) had a logFC greater than or less than 1, containing 191 upregulated and 174 downregulated genes, respectively (Supplementary Materials Table S2: the DEGs of bone metastasis of lung cancer). Combined with the 352 osteolytic metastasis-related targets obtained from the GeneCards (342 targets) and OMIM (10 targets) databases, 698 disease-related targets were filtered after deduplication.

As shown in Figure 1(b), among the 282 GTF-related targets and 698 disease-related targets obtained, 83 intersection targets were found, suggesting that GTF may participate in the treatment of bone metastases through these targets.

### 3.2. Based on the Compound-Target Network of GTF, Key Compounds of Disease Treatment Were Mined

Traditional Chinese medicine is a complex biological network due to its multicomponent and multitarget properties. The network of herb-active compound-potential targets, a useful method to explore key compounds and targets of GTF in the treatment of diseases, was visualized and analyzed by topology, containing 318 nodes and 799 interactions holding 2.274 heterogeneities and 0.486 network centralization, indicating that there were relatively concentrated nodes (key compounds and targets) in the discrete network (Figure 1(c)) [34].

In the treatment of osteolytic metastasis, DX and XX, integral herbs in GTF with 5 and 11 active compounds (accounting for 13.51% and 29.72% of all potential compounds), respectively, directed to 52 and 41 osteolytic metastasis-related targets (accounting for 62.65% and 49.40% of all therapeutic targets), respectively, were considered the most critical traditional Chinese medicines in the treatment of osteolytic metastasis (Figure 1(d)).

To further explore the key pharmacological molecules of GTF in the treatment of osteolytic metastasis, the degree value of each node was calculated, and compounds with a connectivity degree greater than 15 were considered key components in the treatment of diseases. As shown in Figure 1(e), quercetin, kaempferol, rhamnetin, and sesamin may play a more critical role in the treatment of diseases.

### 3.3. Pathways and Key Targets of GTF in the Treatment of Osteolytic Metastasis

**Figure 2:**
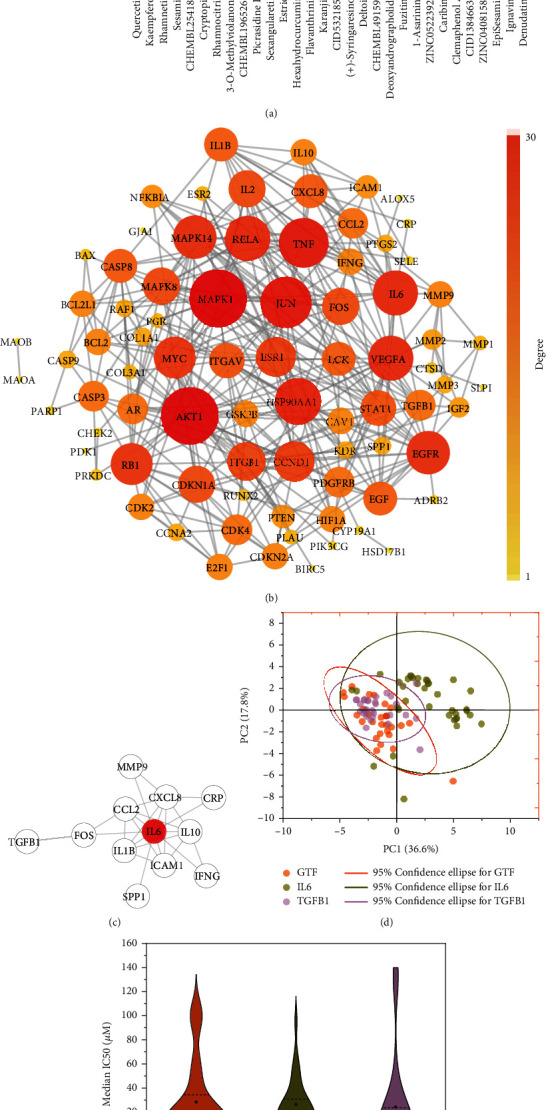
Identification of the biological functions of compounds from GTF in the treatment of osteolytic metastasis. (a) Comprehensive functional characterization of the key compounds. (b) The protein-protein interaction (PPI) of GTF in the treatment of osteolytic metastasis. (c) The kernel of PPI. (d) Principal component analysis of the chemical space of GTF and compounds targeting IL6 and TGFB1. (e) The inhibitory effects of compounds in the GTF and compounds targeting IL6 and TGFB1 on the proliferation of tumor cell lines in public bioactivity databases.

Considering the multicomponent characteristics of GTF in the treatment of diseases, exploring the biological functions of each compound in the treatment of osteolytic metastasis is necessary. In the treatment of osteolytic metastasis, the IL-17, TNF, PI3K-Akt, TGF-beta, and lung cancer signaling pathways showed higher target enrichment results, indicating their potential (Figure 2(a)). By studying the PPI of the intersection targets with a confidence level greater than 0.9, 73 intersection targets and 321 interactions were found (Figure 2(b)), of which IL6 played an important role in the center position. Targeting IL6 may be the crux to the therapeutic effect of GTF (Figure 2(c)).

A chemical space often mentioned in cheminformatics is the space of potential pharmacologically active molecules [28]. PubChem and DrugBank databases were used to screen compounds that can stably bind IL6 and TGFB1, of which 37 and 30 compounds were obtained, respectively. PCA was used for chemical space analysis based on the physicochemical, ADMET, and toxicity properties (Figure 2(d)). The first four principal components captured 73.32% of the variance.

The results showed that the active components contained in GTF had no significant difference from the compounds targeting IL6 and TGFB1 in physicochemical properties and had similar chemical spaces. It also suggested that the compounds contained in GTF may have similar pharmacological activities to those of IL6 and TGFB1.

IC50 and GI50 values were collected from PubChem, DrugBank, ChEMBL, and the literature to investigate the inhibitory effect of compounds from GTF and targeting IL6 and TGFB1 on cell line proliferation. These two metrics were considered the same and were merged in the following analysis.

The calculation results of the median IC50 (or GI50) of the three groups showed that the median IC50 of the GTF and compounds targeting IL6 and TGFB1 were 34.49 *μ*mol, 26.47 *μ*mol, and 24.26 *μ*mol, respectively (*P* = 0.381). Although the compounds from GTF exhibited higher median IC50s than compounds targeting IL6 and TGFB1 in Figure 2(e), there was no significant difference in the inhibitory effects of cell proliferation among the three groups.

### 3.4. GTF Inhibited Bone Destruction by Regulating the Contents of IL6 and TGFB1 In Vivo

**Figure 3:**
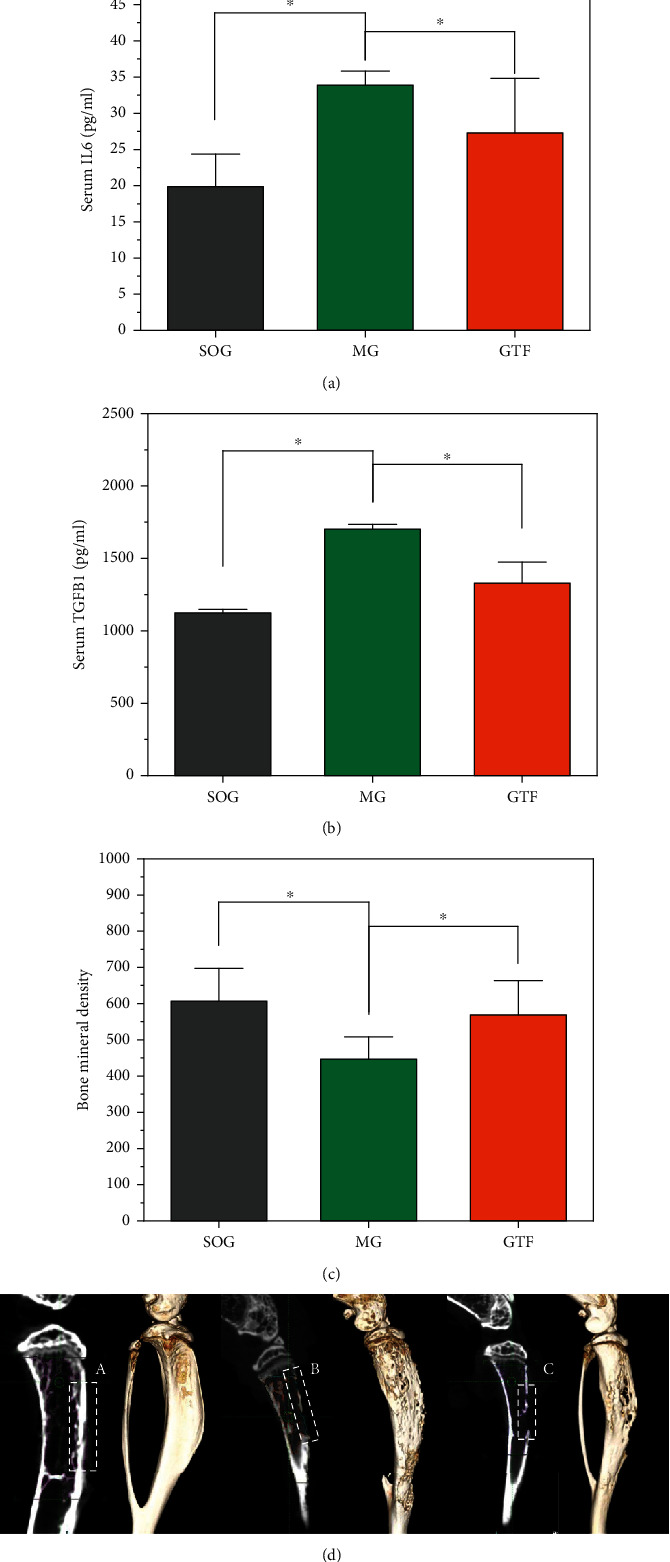
Verification of GTF in alleviating bone destruction in vivo. (a, b) After GTF treatment, serum IL6 and TGFB1 levels were determined by ELISA. Compared with the model group, the serum IL6 and TGFB1 concentrations of GTF were lower. ^∗^*P* < 0.01. (c) BMD was measured after GTF treatment. Compared with the model group, the BMD of the GTF was increased. ^∗^*P* < 0.05. (d) Three-dimensional remodeling of the tumor-bearing tibia (the damaged range of the bone cortex of the SOG, MG, and GTF is represented by A, B, and C, respectively).

In vivo experiments (C57BL/6 mice) were conducted to further understand the effect of GTF on the serum contents of IL6 and TGFB1. The prepared GTF extract (Patent No. 201410415620.1) was applied to the shaved tumor-bearing tibia once a day at a dose of 25 g/kg. After 21 days, blood samples were taken from the orbit, and the serum IL 6 and TGFB1 levels were determined. As shown in Figures 3(a) and 3(b), the serum IL6 contents in SOG, MG, and GTF were 19.86 pg/ml, 33.86 pg/ml, and 27.29 pg/ml, respectively, and the serum TGFB1 contents were 1122.67 pg/ml, 1702.17 pg/ml, and 1300.33 pg/ml, respectively. The experimental results showed that compared with the model group without intervention, the serum IL6 and TGFB1 levels of mice were significantly different (*P* < 0.01).

The BMD was measured to further observe the extent of bone destruction of the tumor-bearing tibia by micro-CT. The results showed a significant increase after the application of GTF (*P* < 0.05) (Figure 3(c)). As shown in Figure 3(f), the 3D reconstructions of the tumor-bearing tibia with (Figure 3(d), C) and without (Figure 3(d), B) the application of GTF showed a difference of the damage extent of cortical bone structure, indicating the GTF had the inhibition of the bone destruction, which could be related to the regulation of the inflammatory factors in bone microenvironment.

### 3.5. Based on Molecular Docking and Dynamics, the Key Compounds Have Been Proven to Stably Bind IL6 and TGFB1

**Figure 4:**
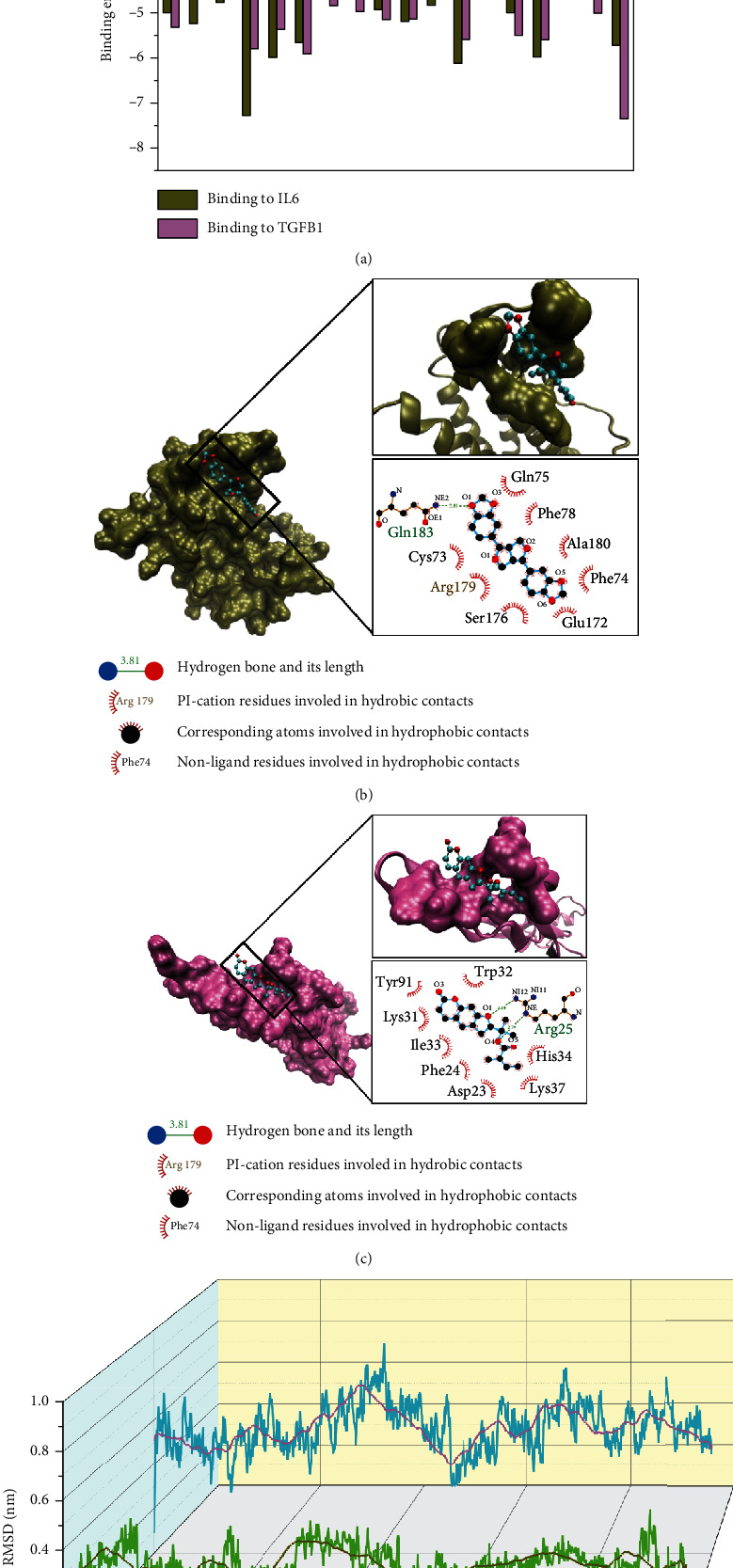
Based on molecular docking and molecular dynamics, the binding stability of key compounds to IL6 and TGFB1 was verified. (a) Binding energy of key compounds. (b) Binding mode of IL6 and sesamin. (c) Binding mode of TGFB1 and deltoin. (d) The root mean square deviation (RMSD) versus time graph of the compounds in the IL6-sesamin and TGFB1-deltoin complexes relative to proteins over 10 ns.

As shown in Figure 4(a), the molecular docking results showed that the docking energies of sesamin and deltoin with IL6 and TGFB1 were -7.28 kcal/mol and -7.35 kcal/mol, respectively, indicating that the binding structures were relatively stable. Further observation of the binding mode between the compound and protein showed that the protein cavity can be occupied by small molecules through hydrophobic interactions, which block the interaction between the active site and the corresponding receptor (Supplementary Materials Figures 1A and 1B). For example, the residues of protein can form hydrophobic interactions with sesamin and deltoin through Cys73, Phe74, Gln75, Phe78, Glu172, Ser176, Arg179, and Alu180 and Asp23, Phe24, Lys31, Trp32, Ile33, His34, Lys37, and Tyr91.

Furthermore, sesamin and deltoin can form hydrogen bonds with IL6 and TGFB1 at residues Gln183 and Arg25, respectively, which made the binding more stable (Figure 4(b) and Figure 4(c)). Moreover, the PI-cation interaction between the Arg179 residue of IL6 and sesamin, where the cation resulted from the hyperconjugated structure of nitrogen-carbon-nitrogen in arginine, can further increase the stability of the binding structure.

Based on the D3Pocket tool, the location and dynamic characteristics of drug-likeness pockets of the receptor were further analyzed. As shown in Supplementary Materials Figure 1C, under dynamic conditions, both purple and orange pockets have drug-forming potential, and the structures of the two pockets disturb each other. After 10 ns of dynamics simulation equilibrium, dynamic cross-correlation (DCC) analysis was further used to evaluate the changes in protein structure mediated by small molecules. The results showed that the Arg179 residue of the IL6-active site was negatively correlated with the movement of the Asp34 residue in the hydrophobic cavity (Supplementary Materials Figure 1D). In contrast, the Arg25 and Arg94 residues of the TGFB1-active site were positively correlated with the residue movement in the arm domain (Supplementary Materials Figure 1E), suggesting that when the drug was bound in the active pocket, it might have a corresponding effect on the movement of residues in the orange pocket.

The structural stability of IL6-sesamin and TGFB1-deltoin complexes was further investigated using molecular dynamics simulations by calculating the root mean square deviation (RMSD) value for positional differences of compounds and proteins. Within 10 ns, the distance between sesamin and the IL6-active pocket was 0.15 nm~0.51 nm, with an average of 0.28 nm. At 3 ns~4 ns, the distance increased briefly and then continued to decrease and reached a peak value of 0.51 nm at 9.39 ns. Compared with the distance between sesamin and IL6, the volatility of deltoin and TGFB1 was significantly increased, the RMSD was between 0.25 nm and 0.94 nm, and the average distance was 0.56 nm. The binding of deltoin to TGFB1 was stable within 2 ns, but the distance increased briefly within 2 ns~4 ns, reached a peak at 4.12 ns, and then continued to decline and fluctuate (Figure 4(d)).

**Figure 5:**
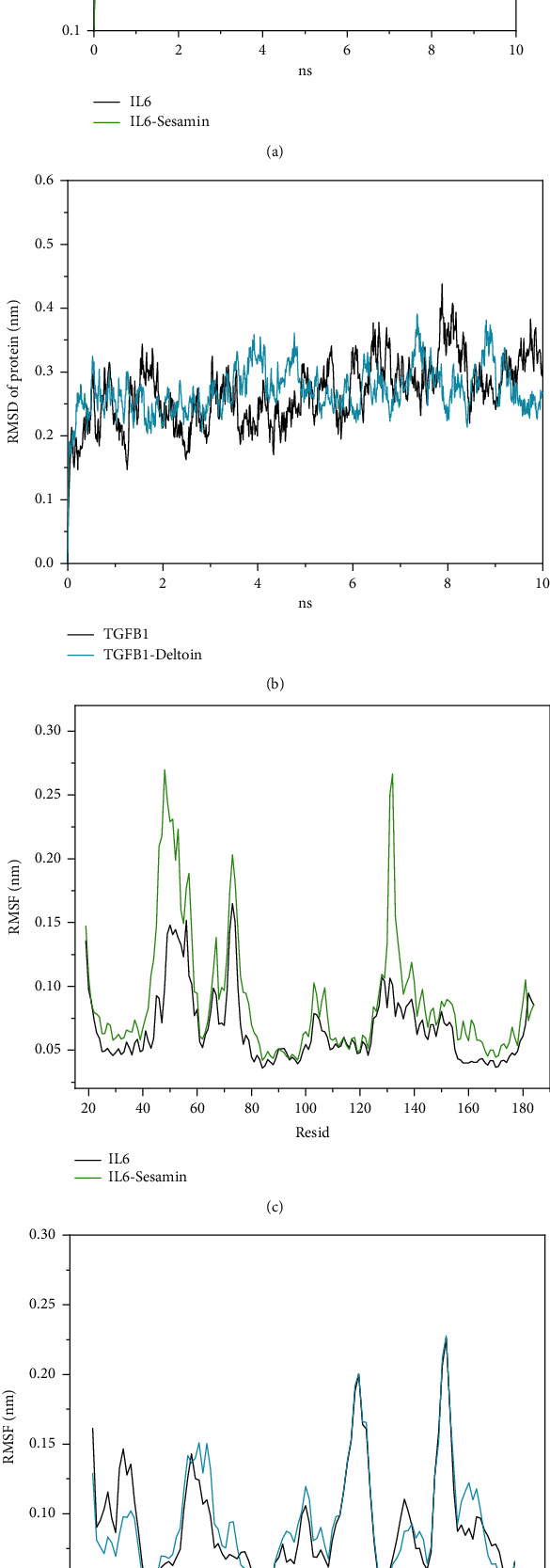
Graphs of RMSD and root mean square fluctuation (RMSF) of IL6-sesamin and TGFB1-deltoin complexes. (a, b) The root mean square deviation (RMSD) versus time graph of the backbone atoms of the IL6-sesamin and TGFB1-deltoin complexes relative to natural proteins over 2 ns. (c) RMSF graph plot of residues of the IL6-sesamin complex (green) relative to residues of IL6 (black) alone. (d) RMSF graph plot of residues of the TGFB1-deltoin complex (blue) relative to residues of TGFB1 (black) alone.

To further explore the fluctuation of small molecular compounds, we analyzed the stability of the protein structure. Compared with the result of the native proteins without ligand, the results of the IL6-sesamin complex after 10 ns molecular dynamics simulations showed greater fluctuation, indicating that the protein structure was more flexible after drug binding (Figure 5(a)). However, compared with native protein, the fluctuation of the backbone of the TGFB1-deltoin complex did not change significantly (Figure 5(b)).

The root mean square fluctuation (RMSF) of residues was calculated to further study the RMSD deviation of the protein before and after sesamin and deltoin binding. The results showed that after sesamin bound to IL6, relatively high flexibility appeared in the ranges of 40-60 and 125-145, which further verified changes in the protein structure, as observed from the RMSD (Figure 5(c)). Although after the combination of deltoin all the residues of TGFB1 showed relatively consistent fluctuation, especially in the range of 66-76 and 91-95, the flexibility of the residues in 50-60 contained in the arm domain was slightly improved (Figure 5(b)).

## 4. Discussion

Transdermal administration has its own advantages because the skin is the largest organ of the human body, and the “first pass effect” is much weaker than that in the liver before reaching systemic circulation [35]. Moreover, the administration is convenient and improves patient compliance.

However, not all ingredients in traditional Chinese medicine can stably penetrate the skin barrier. Drugs with transdermal absorption potential usually have the characteristics of small molecular weight, and the logarithm of the octanol/water partition was between 1 and 4 [21]. The molecular weight of the drug affects the diffusion rate in the skin stratum corneum, approximately following the Stokes-Einstein law [36]. Meanwhile, the transdermal absorption of drugs usually undergoes the distribution process of hydrophilic/lipophilic environments many times, so the relationship between the permeability coefficient of drugs through the skin and the distribution coefficient of octanol/water is usually parabolic [37].

Therefore, according to the screening conditions, 37 compounds with transdermal absorption potential and 282 potential therapeutic targets were obtained and predicted. The complex biological network formed by the interaction between compounds and targets, as well as the interaction between targets, may be the key to the therapeutic effect of GTF.

Bone metastasis is one of the most common complications in advanced patients, with bone destruction and pain as the main manifestations [2]. Previous bioinformatics studies have shown that GTF can regulate cancer-related pain through a variety of biological mechanisms, including bone resorption and remodeling, which are closely related to bone metastasis [11]. This study focused on bone metastasis, and 83 targets of interaction between GTF and bone metastasis were discovered. Through the analysis of the drug-target network, it was found that quercetin, kaempferol, rhamnetin, and sesamin may play a more crucial role in the treatment of diseases. All of the above compounds were natural phytochemicals and had clear anticancer effects.

Studies have shown that quercetin and kaempferol can exert anticancer effects through a variety of mechanisms, including cell apoptosis by mediating lysosome activation and ferroptosis [38, 39], inhibiting invasion and metastasis by restraining epithelial-mesenchymal transition [40, 41], blocking the cell cycle, and inhibiting cancer cell proliferation [42, 43].

Further analysis of the intersecting targets showed that the targets were mainly enriched in the IL-17, TNF, PI3K-Akt, and TGF-beta signaling pathways. Studies have shown that osteoclast activation is mainly regulated by RANKL, which is one of the necessary conditions for osteolytic metastasis [44, 45]. RANKL upregulation by TNF-*α* promotes osteoclast maturation and differentiation through the PI3K-Akt signaling pathway [46, 47]. Meanwhile, TNF-*α* is one of the strongest inducers of bone resorption and can stimulate the expression of IL-17, which is considered an indirect stimulator of osteoclasts, destroying the balance of RANKL/OPG, eventually leading to bone destruction [48, 49]. TGF-*β* has dual effects, which suppress tumor growth at a nearly stage and promote invasion and metastasis to bones in the late stage. The targets of active compounds were enriched in the above pathways, indicating that GTF may achieve bone protection through the interaction between those pathways.

In the present research, the biological mechanism of GTF in the treatment of osteolytic bone metastasis was further explored. As expected, the mechanism is multitarget and multichannel. The method of PPI combined with MCODE was used to further screen the nodes that are at the core and play a more critical role in the multitarget. The results showed that IL6 was the core of the PPI network and connected with TGFB1 through the Fos factor. Regulating IL6 may be the key to the treatment of bone metastasis by GTF. As an inflammatory factor, IL6 is produced by tumor cells in the bone microenvironment through autocrine and paracrine. It can interact with the IL6 receptor (IL6R) and is a key regulator of bone remodeling [5]. Studies have shown that IL6 can promote the formation of osteoclasts by binding to the IL6 receptor (IL6R) and ultimately lead to bone destruction. Similarly, an anti-IL6R antibody can prevent bone metastasis in animal models [4, 50]. In addition, blocking PI3K/AKT, as the downstream pathway activated by IL6, can inhibit the differentiation of osteoclasts induced by lung cancer cells or interleukins [51], which highlights the important role of IL6 in bone homeostasis. Meanwhile, IL6 also plays an important role in acute inflammation and pain [52]. Therefore, our study suggests that GTF may restrict the damage scope of bone by reducing the related inflammatory response of tumors in the bone microenvironment.

TGFB1 stimulation enhanced the recruitment and activation of SMAD and played a role in regulating cell proliferation, differentiation, and growth. Under normal circumstances, TGFB1, as an effective stimulant of osteoblasts, can cause osteoblast proliferation and differentiation [53]. Different concentrations of TGFB1 play different biological roles in the regulation of osteoclast differentiation. Studies have shown that TGFB1 can promote or inhibit osteoclast differentiation at low and high doses, respectively. The decrease in TGFB1 levels was related to the increase in bone mass [54]. Compared with the direct involvement of IL6 in the process of bone destruction, TGFB1 mainly plays a role in the process of tumor bone metastasis. The results showed that the serum TGFB1 level and the activity of the TGFB1 signaling pathway were significantly increased in tumor patients with bone metastases [55]. Moreover, TGFB1 was involved in the whole process of tumor migration, bone colonization, dormancy, and bone resorption and remodeling. The interaction of the IL6 and TGFB1 signaling pathways plays an important role in SREs caused by tumor escape, bone colonization, and bone destruction. Therefore, IL6 and TGFB1 were selected as targets for follow-up studies. The results of chemical space showed that the active compounds had similar pharmacological activities to drugs that target IL6 and TGFB1, and there was little difference in IC50 between them.

Animal experiments further verified the effect of GTF on serum IL6 and TGFB1, showing that GTF can effectively reduce the content of serum IL6 and TGFB1. Due to the similar physical and chemical properties and toxicity with IL6 and TGFB1, the result is not unexpected. IL6 and TGFB1 were regarded as potential targets of GTF in the treatment of osteolytic bone destruction. The micro-CT results showed that compared with the MG, the BMD and bone destruction range of the GTF group were improved and limited. GTF treatment can reduce the content of IL6 in serum, improve the microenvironment of tumors and bones, increase bone density, reduce the range of bone cortex damage, and achieve bone protection. However, it is undeniable that IL6 and TGFB1 were not the only factors for the therapeutic effects of GTF, and further study on the multitarget and multichannel mechanism is still necessary.

To make ligands and receptors more effectively bind and play a role, compounds were expected to occupy the active pocket of proteins and interact with key residues of proteins. Research showed that among the active compounds, sesamin and deltoin anchored in the largest central cavity of the protein and formed spontaneous binding through hydrophobic forces, van der Waals forces, and hydrogen bonds, which were considered to be the most stable compounds binding to IL6 and TGFB1, respectively. Molecular dynamics studies showed that within 10 ns, the binding of sesamin to IL6 was more stable and increased the flexibility of IL6 residues.

RMSF also showed that when sesamin combined with the active pocket of IL6, although the fluctuation of the Arg179 residue was not obvious, the distal Arg34 showed high flexibility, and the cavity containing Arg34 was mainly used to form an interaction force with the Phe169 residue at the Gp130 receptor IIA site, which played a crucial role in the process of Gp130 recognizing IL6 and activating. Meanwhile, Arg179 in the active site was one of the three conserved antigen epitopes of IL6, which can form an interaction with the Phe229 site of IL6R*α*, thus promoting the formation of an effective signal complex between sites II and III with the immunoglobulin activation domain of IL6R*α* successively [56]. The compound sesamin contained in GTF can stably occupy the site I of IL6 and interact with Arg179. At the same time, it affected the flexibility of the distal Arg34 residue impacting the formation of a complex with the receptor and blocking downstream signal transmission, thus playing a role in alleviating bone damage.

Similarly, after deltoin was combined with TGFB1, the flexibility of the residues of the arm domain was improved to a certain extent, and the arm domain was used to bind to integrin families. The integrin signaling pathway has been proven to be involved in the construction of the bone microenvironment and the process of tumor migration. In addition, TGFB1 binding to the type II receptor (T*β*RII) transmitted signals downstream. Compared with another cytokine of the TGF*β* family, the active domain of TGFB1 can interact with T*β*RII stably and distinctively. This feature was that the Ile53 residue of T*β*RII can fill the hydrophobic cavity inside TGFB1. The Arg25 and Arg94 residues on the active domain of TGFB1 can interact with Asp32 and Glu119 on T*β*RII. The binding energy formed by the two arginine residues accounts for more than 30% of the total binding energy. Arg25 and Arg94 in the hydrophobic cavity of TGFB1 played an important role in the stable binding and signal transmission with T*β*RII [57]. Deltoin occupied the cavity and formed hydrogen bonds at Arg25 residues, which hindered the binding of TGFB1 with T*β*RII, thus blocking signal transmission.

Therefore, the key pharmacodynamic molecules of GTF, sesamin and deltoin, which occupy the active site of the protein, and interfere with the distal domain, hindered the normal function of the protein. However, although deltoin can form hydrogen bonds with TGFB1, compared with sesamin, the fluctuation of deltoin combined with TGFB1 is significantly greater than that of sesamin and reaches the peak at 4.12 ns, with a peak of 0.94 nm. It is suggested that although the molecular docking results show that deltoin can spontaneously bind to the cavity of TGFB1, the binding is not as stable as expected, and the binding ability of the complex still needs to be further studied. Here, our study is cautious about the interpretation of the binding ability of deltoin to TGFB1.

## 5. Conclusion

Inflammation is closely related to oncogenesis, and “inflammation-carcinogenesis” is also involved in the process of tumor bone metastasis and bone destruction. Traditional alternative therapies represented by traditional Chinese medicine have been proven to play an important role in regulating immunity and inflammation. This work shows that the compounds in GTF can play a role in bone protection by stably combining with IL6 and TGFB1, which may also be one of the reasons that GTF can prophylax and treat cancer-related pain.

Among the active compounds, sesamin and deltoin have the potential to become anti-inflammatory and anticancer drugs. The reasons for the therapeutic effect of GTF are intricate, comprehensive, multimechanism, and multichannel. Herein, we found that GTF can exert its pharmacological effect by affecting the IL-17, TNF-*α*, PI3K-Akt, and TGF-*β* pathways, laying a foundation for further research in the future. Note that, as an external clinical preparation, GTF may have a synergistic effect in the future treatment of bone metastasis combined with zoledronic acid, providing a new idea for the treatment of bone metastasis and cancer pain.

## Data Availability

The data used to support the findings of this study are included within the article and the supplementary information files.

## Supplementary Materials

Supplementary Materials Table S1: the potential transdermal absorption compounds of GTF. Table S2: the GEO database was used for the analysis of differentially expressed genes (DEGs) in bone metastasis of lung cancer. Figure S1: dynamic cross-correlation between active sites and allosteric sites.
