# Supplementary material for: The Molecular Mechanism of Traditional Chinese Medicine Prescription: Gu-tong Formula in Relieving Osteolytic Bone Destruction
Source: Biomed Res Int. 2022 Jul 13;2022:4931368. doi: 10.1155/2022/4931368 (PMC9300326; doi:10.1155/2022/4931368)
Supplement: Supplementary Materials — Table S1: the potential transdermal absorption compounds of GTF. Table S2: the GEO database was used for the analysis of differentially expressed genes (DEGs) in bone metastasis of lung cancer. Figure S1: dynamic cross-correlation between active sites and allosteric sites. [file 4931368.f1.docx]

**Supporting Information**

The molecular mechanism of traditional Chinese medicine prescription - Gu-tong formula in relieving osteolytic bone destruction

*Chang Jinyuana, Jiang Zhenglonga, Jin Weia, Wang Yaohanb, Li Jiea, Chen Jiayanga, Li Haoc, Feng Lia,**

*a National Cancer Center/National Clinical Research Center for Cancer/Cancer Hospital, Chinese Academy of Medical Sciences and Peking Union Medical College, Beijing (100021), China*

*b Department of oncology, Beijing hospital of integrated traditional Chinese and Western Medicine, Beijing (100039), China*

*c Beijing University of Chinese Medicine, Beijing (100029), China*

** Correspondence: fengli663@126.com; Tel.: +86-010-877-880-30*

**Dynamic cross-correlation between active sites and allosteric sites.**

Binding patterns of sesamin and deltoin with IL6 and TGFB1, respectively (**supplementary Fig. A and B**). Sesamin and deltoin occupied the active pocket and affected the binding of proteins to their receptors.

The active site and allosteric site of the protein (**supplementary Fig. C**). Purple pockets were used to bind with small molecular compounds.

Dynamic cross-correlation analysis between residues (**supplementary Fig. D and E**).

**The prediction of active compounds and potential targets of GTF and osteolytic metastasis by database screening and DEG analysis**

The compounds with transdermal absorption potential and their molecular formulars were screened and obtained by the TCMSP database and PubChem database, respectively. The potential targets were predicted by the TCMSP, ChEMBL and ChemMapper databases. Note that GTF contains 9 kinds of traditional Chinese medicine with complex chemical components. Through database screening and prediction, 37 potential compounds (**Table S1**) and 282 potential therapeutic targets were obtained for further analysis.

The potential transdermal absorption compounds of GTF are listed in the **Table S1**.

The GEO database was used for the analysis of differentially expressed genes (DEGs) in bone metastasis of lung cancer. As shown in **Table S2**, 365 targets (out of 928 gene targets with a P value less than 0.05) had a logFC greater than or less than 1, containing 191 upregulated and 174 downregulated genes, respectively.

The DEGs of bone metastasis of lung cancer are listed in the **Table S2**.


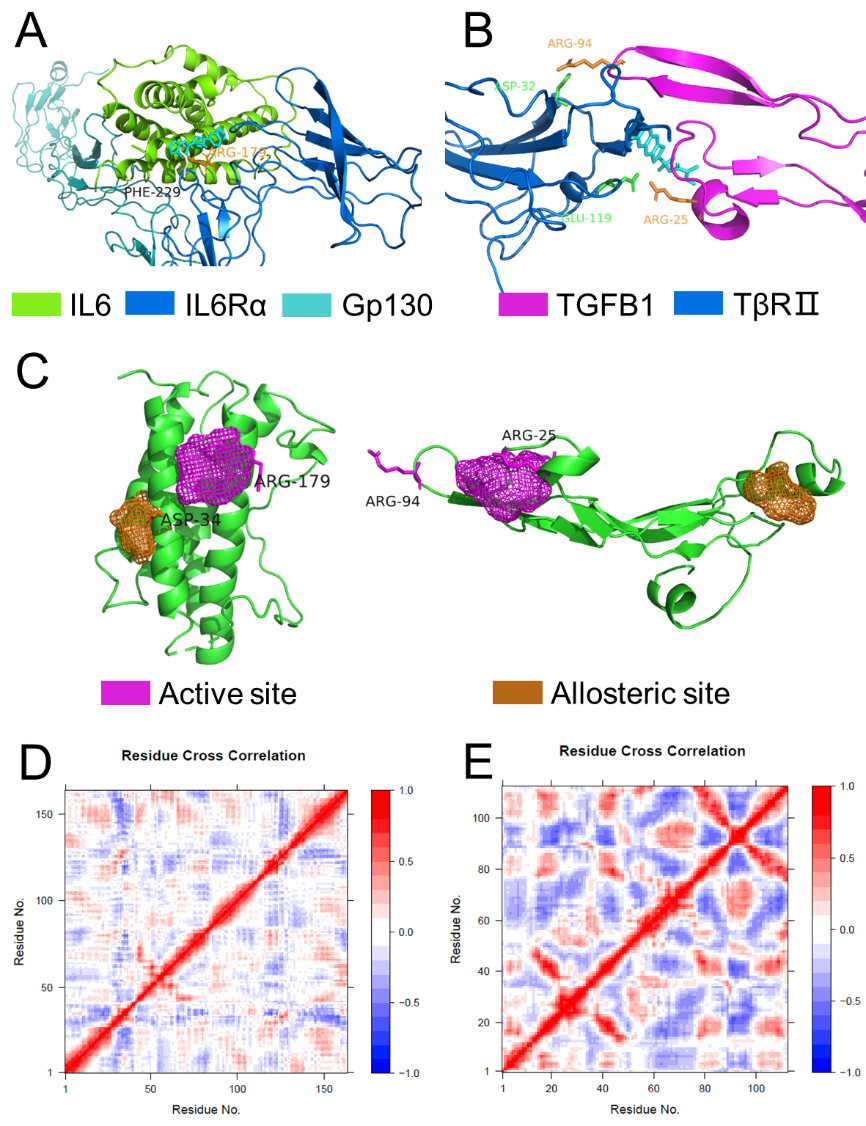


**Supplementary Fig.** Dynamic cross-correlation between active sites and allosteric sites.

**Table S1.** GTF contains drugs with transdermal absorption potential

| Latin name | Molecule Name | MW | AlogP | DL |
| --- | --- | --- | --- | --- |
| Radix Aconiti Lateralis Preparata | CHEMBL361568 | 332.48 | 2.1 | 0.32 |
| Radix Aconiti Lateralis Preparata | Deltoin | 328.39 | 2.48 | 0.37 |
| Radix Aconiti Lateralis Preparata | Deoxyandrographolide | 334.5 | 3.02 | 0.31 |
| Radix Aconiti Lateralis Preparata | Karanjin | 292.3 | 2.94 | 0.34 |
| Radix Aconiti Lateralis Preparata | benzoylnapelline | 463.67 | 3.12 | 0.53 |
| Radix Aconiti Lateralis Preparata | denudatine | 343.56 | 2.25 | 0.67 |
| Radix Aconiti Lateralis Preparata | fuzitine | 342.45 | 3.12 | 0.54 |
| Radix Aconiti Lateralis Preparata | (R)-Norcoclaurine | 271.34 | 2.57 | 0.21 |
| Radix Aconiti Lateralis Preparata | ignavine | 449.59 | 1.22 | 0.25 |
| Radix Aconiti Lateralis Preparata | Delavaconitine | 497.69 | 1.38 | 0.37 |
| Rhizoma Curculiginis | (+)-Syringaresinol | 418.48 | 2.1 | 0.72 |
| Rhizoma Curculiginis | CID5321858 | 332.33 | 2.6 | 0.28 |
| Rhizoma Curculiginis | CHEMBL1965265 | 316.33 | 2.9 | 0.33 |
| Rhizoma Curculiginis | yuccagenin | 430.69 | 3.67 | 0.79 |
| Herba Asari | Picrasidine D | 254.31 | 3.38 | 0.19 |
| Herba Asari | Caribine | 326.43 | 1.22 | 0.83 |
| Herba Asari | Estriol | 288.42 | 2.85 | 0.36 |
| Herba Asari | Cryptopin | 369.45 | 3.15 | 0.72 |
| Herba Asari | sesamin | 354.38 | 2.24 | 0.83 |
| Herba Asari/Rhizoma Zingiberis | SCHEMBL119969 | 360.49 | 3.68 | 0.31 |
| Herba Asari | 3-O-Methylviolanone | 330.36 | 2.67 | 0.33 |
| Herba Asari/Flos Caryophylli | kaempferol | 286.25 | 1.77 | 0.24 |
| Herba Asari | ZINC05223929 | 354.38 | 2.24 | 0.83 |
| Herba Asari | Episesamin | 354.38 | 2.24 | 0.83 |
| Herba Asari | 1-Asarinine | 354.38 | 2.24 | 0.83 |
| Rhizoma Zingiberis | hexahydrocurcumin | 374.47 | 3.31 | 0.41 |
| Rhizoma Zingiberis | ZINC04081584 | 302.5 | 3.34 | 0.39 |
| Rhizoma Zingiberis | CHEMBL491591 | 344.44 | 3.33 | 0.32 |
| Rhizoma Zingiberis | Sexangularetin | 316.28 | 1.76 | 0.3 |
| Radix Clematidis | clemaphenol A | 358.42 | 2.13 | 0.52 |
| Pseudobulbus Cremastrae Seu Pleiones | CHEMBL254186 | 242.29 | 3.4 | 0.18 |
| Pseudobulbus Cremastrae Seu Pleiones | flavanthrinin | 240.27 | 3.1 | 0.18 |
| Flos Caryophylli | Rhamnocitrin | 300.28 | 2.02 | 0.27 |
| Flos Caryophylli | CID13846639 | 318.35 | 1.76 | 0.3 |
| Flos Caryophylli | Rhamnetin | 316.28 | 1.76 | 0.3 |
| Flos Caryophylli | quercetin | 302.25 | 1.5 | 0.28 |
| Scorpio | CID5318035 | 418.58 | 1.91 | 0.65 |

**Table S2.** DEGs of bone metastasis of lung cancer

| Gene symbol | logFC | P.Value | adj.P.Val | change |
| --- | --- | --- | --- | --- |
| IL32 | -2.2011 | 1.71E-05 | 0.094982 | DOWN |
| MLLT11 | 1.829142 | 1.92E-05 | 0.094982 | UP |
| TCF4 | 1.784995 | 2.53E-05 | 0.094982 | UP |
| CREM | 1.224681 | 3.22E-05 | 0.094982 | UP |
| LCN2 | -3.42302 | 4.60E-05 | 0.102225 | DOWN |
| FAM46A | 1.161566 | 5.20E-05 | 0.102225 | UP |
| GRAMD3 | -1.80681 | 7.32E-05 | 0.12354 | DOWN |
| NR0B1 | 2.090053 | 9.49E-05 | 0.134892 | UP |
| RAPGEF4 | 1.796128 | 0.000103 | 0.134892 | UP |
| NR4A3 | 1.202306 | 0.000165 | 0.180669 | UP |
| INHBA | 1.564535 | 0.00021 | 0.180669 | UP |
| MSLN | -2.28761 | 0.000223 | 0.180669 | DOWN |
| GRAMD1B | 1.689303 | 0.000225 | 0.180669 | UP |
| PYCARD | -2.33029 | 0.00023 | 0.180669 | DOWN |
| TOX3 | -1.44158 | 0.000231 | 0.180669 | DOWN |
| PDE4B | 1.630629 | 0.000245 | 0.180669 | UP |
| AMOTL2 | -1.66856 | 0.000263 | 0.180669 | DOWN |
| CHRNA9 | 3.079848 | 0.000275 | 0.180669 | UP |
| TRPV2 | 1.865337 | 0.000298 | 0.184277 | UP |
| PPFIBP2 | -1.59522 | 0.000321 | 0.184277 | DOWN |
| QPCT | 2.444876 | 0.00033 | 0.184277 | UP |
| C1orf106 | -1.64574 | 0.000343 | 0.184277 | DOWN |
| OLFM4 | -1.02659 | 0.000379 | 0.186785 | DOWN |
| PDZK1IP1 | -2.72835 | 0.00038 | 0.186785 | DOWN |
| XK | -1.97748 | 0.000397 | 0.187408 | DOWN |
| COL4A6 | 2.035798 | 0.000413 | 0.187503 | UP |
| LRRTM2 | -1.7027 | 0.000463 | 0.200395 | DOWN |
| ALDH2 | -1.37936 | 0.000497 | 0.200395 | DOWN |
| FGF13 | 2.001587 | 0.000525 | 0.200395 | UP |
| ALOX5 | -1.05328 | 0.000537 | 0.200395 | DOWN |
| SALL1 | 2.583099 | 0.000538 | 0.200395 | UP |
| FLRT3 | -1.12679 | 0.000546 | 0.200395 | DOWN |
| GAP43 | 1.271961 | 0.000614 | 0.201557 | UP |
| OPN3 | 1.326527 | 0.000624 | 0.201557 | UP |
| LAMP3 | -1.9322 | 0.000649 | 0.201557 | DOWN |
| SMYD3 | 1.147106 | 0.000651 | 0.201557 | UP |
| RALGPS2 | -1.11777 | 0.000656 | 0.201557 | DOWN |
| LSR | -1.63038 | 0.000668 | 0.201557 | DOWN |
| T | 2.576902 | 0.000683 | 0.201557 | UP |
| EPPK1 | -1.01612 | 0.0007 | 0.201557 | DOWN |
| GPR37 | 1.555479 | 0.00073 | 0.205107 | UP |
| SEMA3A | 1.952652 | 0.000872 | 0.212902 | UP |
| RTN1 | 2.479711 | 0.000889 | 0.212902 | UP |
| NRCAM | 1.376295 | 0.0009 | 0.212902 | UP |
| PZP | 1.128503 | 0.000918 | 0.212902 | UP |
| SCG2 | 2.109372 | 0.000938 | 0.212902 | UP |
| CTSH | -1.98633 | 0.000942 | 0.212902 | DOWN |
| SCG5 | -1.11091 | 0.000943 | 0.212902 | DOWN |
| FBLN2 | -1.71429 | 0.000946 | 0.212902 | DOWN |
| NUPR1 | -1.38609 | 0.000956 | 0.212902 | DOWN |
| BDKRB2 | -1.63831 | 0.000961 | 0.212902 | DOWN |
| TLL1 | -1.11154 | 0.000974 | 0.212902 | DOWN |
| NTS | 2.826658 | 0.001011 | 0.214509 | UP |
| NRN1 | 1.630833 | 0.001094 | 0.214509 | UP |
| PDE1C | 1.437905 | 0.001113 | 0.214509 | UP |
| PDE3A | 1.356472 | 0.001179 | 0.214509 | UP |
| HLA-DRA | -2.49453 | 0.001183 | 0.214509 | DOWN |
| XAF1 | -1.04333 | 0.001186 | 0.214509 | DOWN |
| UCHL1 | 1.178682 | 0.001187 | 0.214509 | UP |
| APOL1 | -1.83603 | 0.001205 | 0.214509 | DOWN |
| TSPAN7 | 1.661534 | 0.001216 | 0.214509 | UP |
| BST2 | -1.90481 | 0.001227 | 0.214509 | DOWN |
| CRLF2 | 1.457104 | 0.00123 | 0.214509 | UP |
| PART1 | 1.334074 | 0.001235 | 0.214509 | UP |
| DEPDC1 | 1.126227 | 0.001241 | 0.214509 | UP |
| PROCR | 1.197883 | 0.001257 | 0.214509 | UP |
| ANKRD1 | -1.26904 | 0.001279 | 0.214509 | DOWN |
| SDC4 | -1.22956 | 0.00129 | 0.214509 | DOWN |
| TMEM100 | 2.798081 | 0.001362 | 0.223264 | UP |
| PAK3 | 2.001663 | 0.001431 | 0.231429 | UP |
| MAN1C1 | -1.08173 | 0.001476 | 0.232536 | DOWN |
| RARRES1 | -2.1534 | 0.001493 | 0.232536 | DOWN |
| DTX4 | -1.47016 | 0.001529 | 0.232536 | DOWN |
| PTPN20B | 2.407187 | 0.001534 | 0.232536 | UP |
| RARRES2 | -1.90036 | 0.001541 | 0.232536 | DOWN |
| RGS2 | 1.873367 | 0.001556 | 0.232536 | UP |
| FCGRT | -1.8489 | 0.001581 | 0.233373 | DOWN |
| IRGC | -1.02321 | 0.001626 | 0.233632 | DOWN |
| SLC44A1 | 1.144609 | 0.001658 | 0.233632 | UP |
| BICC1 | -1.38384 | 0.001662 | 0.233632 | DOWN |
| RPE65 | 1.371971 | 0.001695 | 0.235427 | UP |
| EYA4 | 1.433217 | 0.001728 | 0.237239 | UP |
| TMPRSS3 | -1.5142 | 0.001819 | 0.243532 | DOWN |
| PNMA2 | -1.2001 | 0.001836 | 0.243532 | DOWN |
| NID2 | 2.4563 | 0.001875 | 0.243786 | UP |
| MEF2C | 1.500401 | 0.0019 | 0.243786 | UP |
| CTSZ | 1.026387 | 0.001936 | 0.245823 | UP |
| MMP7 | -1.4487 | 0.001997 | 0.250801 | DOWN |
| DLG2 | 1.565905 | 0.002032 | 0.252504 | UP |
| LCE2B | 1.000851 | 0.002096 | 0.256297 | UP |
| SOAT2 | 1.048643 | 0.002106 | 0.256297 | UP |
| LINC00260 | -1.02597 | 0.002165 | 0.260809 | DOWN |
| ANKRD2 | -1.19635 | 0.002201 | 0.262449 | DOWN |
| GPR97 | 1.744858 | 0.002249 | 0.262528 | UP |
| SEMA3D | 1.660583 | 0.002266 | 0.262528 | UP |
| EPHA2 | -1.17971 | 0.002268 | 0.262528 | DOWN |
| LOC100506699 | 1.489873 | 0.002434 | 0.278068 | UP |
| CABYR | 1.428451 | 0.00245 | 0.278068 | UP |
| HIST1H2BN | 1.158997 | 0.002486 | 0.279537 | UP |
| SLC43A3 | -1.83182 | 0.002526 | 0.281394 | DOWN |
| CYTIP | 1.194443 | 0.002614 | 0.288379 | UP |
| C4orf19 | -1.25241 | 0.002727 | 0.288725 | DOWN |
| SPP1 | 1.523896 | 0.002734 | 0.288725 | UP |
| GABBR2 | 2.027265 | 0.002771 | 0.288725 | UP |
| F2RL2 | 1.12449 | 0.002792 | 0.288725 | UP |
| SPAG4 | 1.105126 | 0.002861 | 0.288725 | UP |
| G6PD | 1.082371 | 0.00288 | 0.288725 | UP |
| GRAMD1C | -1.4989 | 0.002881 | 0.288725 | DOWN |
| HPGDS | 1.08883 | 0.002904 | 0.288725 | UP |
| CAT | -1.1113 | 0.002917 | 0.288725 | DOWN |
| SLCO3A1 | -1.05143 | 0.00295 | 0.288725 | DOWN |
| ARHGEF5 | -1.43387 | 0.002961 | 0.288725 | DOWN |
| ABCC2 | 1.692054 | 0.003005 | 0.288725 | UP |
| LY75 | -1.89181 | 0.003035 | 0.288725 | DOWN |
| MARCKS | -1.44345 | 0.003116 | 0.288725 | DOWN |
| BIK | -1.88258 | 0.003153 | 0.288725 | DOWN |
| CXCL5 | 1.861494 | 0.003155 | 0.288725 | UP |
| BFSP1 | 1.058335 | 0.00318 | 0.288725 | UP |
| PLA2G3 | 1.104164 | 0.003188 | 0.288725 | UP |
| NEFL | 2.496531 | 0.003211 | 0.288725 | UP |
| NTRK1 | 1.119269 | 0.00326 | 0.288725 | UP |
| CYP7A1 | 1.200507 | 0.003265 | 0.288725 | UP |
| KCNE4 | 2.012241 | 0.003299 | 0.288725 | UP |
| NR4A2 | 1.215919 | 0.003356 | 0.288725 | UP |
| ALS2CL | -1.09936 | 0.003374 | 0.288725 | DOWN |
| PLEKHA6 | -1.54282 | 0.003374 | 0.288725 | DOWN |
| TNFAIP3 | 1.6103 | 0.00344 | 0.288725 | UP |
| MGAT4A | -1.1474 | 0.003445 | 0.288725 | DOWN |
| ALDOC | 1.298144 | 0.003456 | 0.288725 | UP |
| PITPNC1 | 1.303412 | 0.003473 | 0.288725 | UP |
| SUSD5 | 1.168228 | 0.003506 | 0.289476 | UP |
| MST1R | -1.31437 | 0.003647 | 0.296923 | DOWN |
| DNASE2B | 1.039124 | 0.003686 | 0.298022 | UP |
| ADRB2 | -1.35576 | 0.003757 | 0.301775 | DOWN |
| SERPIND1 | 3.270763 | 0.003852 | 0.307263 | UP |
| ZNF331 | -1.06749 | 0.003928 | 0.311205 | DOWN |
| TNFAIP6 | 1.337427 | 0.003974 | 0.312772 | UP |
| HSPB3 | 1.752437 | 0.004028 | 0.312874 | UP |
| TBX2 | 1.261851 | 0.00406 | 0.313295 | UP |
| SOX9 | 1.362687 | 0.004137 | 0.315113 | UP |
| KCNJ2 | -1.83029 | 0.004165 | 0.315232 | DOWN |
| CALY | 1.108711 | 0.004242 | 0.318855 | UP |
| VIP | -1.05113 | 0.004318 | 0.318855 | DOWN |
| FARP1 | -1.07077 | 0.004327 | 0.318855 | DOWN |
| RARRES3 | -1.64081 | 0.004378 | 0.318855 | DOWN |
| CA2 | 2.103306 | 0.004484 | 0.318855 | UP |
| KIF21B | -1.52611 | 0.004497 | 0.318855 | DOWN |
| CUBN | 1.109504 | 0.004531 | 0.318855 | UP |
| BCAS3 | 1.036838 | 0.00457 | 0.318855 | UP |
| EDNRB | 1.800478 | 0.004603 | 0.318855 | UP |
| C1orf116 | -1.59311 | 0.004618 | 0.318855 | DOWN |
| PLAG1 | -1.25121 | 0.004676 | 0.319091 | DOWN |
| ABCG2 | 1.166906 | 0.004872 | 0.328656 | UP |
| SPINT2 | -2.5443 | 0.004908 | 0.329243 | DOWN |
| GJA1 | 1.507509 | 0.005001 | 0.331679 | UP |
| UTS2 | -1.10924 | 0.005197 | 0.335683 | DOWN |
| COLEC10 | 1.522357 | 0.005303 | 0.335683 | UP |
| PCK1 | 1.232722 | 0.005322 | 0.335683 | UP |
| CHL1 | 1.283925 | 0.005394 | 0.335683 | UP |
| PRKAA2 | 1.02271 | 0.005396 | 0.335683 | UP |
| CD24 | -1.66781 | 0.005441 | 0.335683 | DOWN |
| COL5A2 | 2.153971 | 0.005448 | 0.335683 | UP |
| THBD | 1.051288 | 0.005474 | 0.335683 | UP |
| SPARC | -1.66455 | 0.00554 | 0.335683 | DOWN |
| HCP5 | -1.77021 | 0.005552 | 0.335683 | DOWN |
| PLLP | -1.18235 | 0.005561 | 0.335683 | DOWN |
| RHOH | 1.450103 | 0.005755 | 0.344863 | UP |
| CHST7 | 1.450312 | 0.005969 | 0.355247 | UP |
| STS | -1.0177 | 0.006167 | 0.358094 | DOWN |
| POPDC3 | 1.196331 | 0.006366 | 0.361543 | UP |
| RPS4Y1 | 2.484725 | 0.006367 | 0.361543 | UP |
| DSP | -2.39439 | 0.00637 | 0.361543 | DOWN |
| ITGBL1 | 1.055837 | 0.00648 | 0.365339 | UP |
| C1orf54 | 1.330094 | 0.00656 | 0.365339 | UP |
| ELF3 | -1.25762 | 0.006729 | 0.370658 | DOWN |
| TNFRSF21 | -1.21302 | 0.006729 | 0.370658 | DOWN |
| CDH19 | 1.511012 | 0.006754 | 0.370658 | UP |
| CDKN2A | -1.17533 | 0.006781 | 0.370658 | DOWN |
| CDCP1 | -1.00965 | 0.00683 | 0.371004 | DOWN |
| SLC2A3 | 1.129131 | 0.007179 | 0.379783 | UP |
| RFK | 1.128477 | 0.007248 | 0.380332 | UP |
| RNF39 | -1.05068 | 0.007296 | 0.381122 | DOWN |
| ISG15 | -1.39516 | 0.007603 | 0.38771 | DOWN |
| ARID3B | 1.172792 | 0.007619 | 0.38771 | UP |
| CD1C | 1.014821 | 0.007717 | 0.390174 | UP |
| GREM1 | -1.22202 | 0.007758 | 0.390174 | DOWN |
| CLU | 1.003973 | 0.008148 | 0.405469 | UP |
| PAEP | 1.677208 | 0.008682 | 0.425145 | UP |
| CHI3L1 | -1.48966 | 0.008715 | 0.425145 | DOWN |
| ACSL5 | -1.37655 | 0.009065 | 0.428605 | DOWN |
| EDN1 | -1.40482 | 0.009163 | 0.428605 | DOWN |
| PRSS8 | -1.39671 | 0.009312 | 0.428605 | DOWN |
| CGREF1 | 1.12362 | 0.009356 | 0.428605 | UP |
| CPS1 | 1.991784 | 0.00936 | 0.428605 | UP |
| PDE1A | 1.360748 | 0.009366 | 0.428605 | UP |
| FAM155A | 1.122928 | 0.009413 | 0.429072 | UP |
| MAGEB2 | 1.859004 | 0.009669 | 0.435692 | UP |
| GPR87 | -1.60012 | 0.009813 | 0.440271 | DOWN |
| LCN1 | 1.490378 | 0.009849 | 0.440271 | UP |
| DGKD | 1.006369 | 0.009897 | 0.440271 | UP |
| CD36 | 1.928982 | 0.009973 | 0.440271 | UP |
| NUDT11 | 1.364265 | 0.010208 | 0.440372 | UP |
| PCSK5 | 1.463233 | 0.010372 | 0.441375 | UP |
| OLFML2A | 2.219746 | 0.010446 | 0.441375 | UP |
| HLA-DMA | -1.09003 | 0.010504 | 0.441375 | DOWN |
| PRRG4 | -1.11464 | 0.010752 | 0.442298 | DOWN |
| PLBD1 | -1.58496 | 0.010898 | 0.444703 | DOWN |
| CXCL6 | -1.27486 | 0.010926 | 0.444703 | DOWN |
| ADRA2A | 1.062752 | 0.010967 | 0.444703 | UP |
| ZNF804A | 1.175546 | 0.011602 | 0.457294 | UP |
| DENND2D | -1.0035 | 0.011815 | 0.458793 | DOWN |
| TMEM176A | -1.27428 | 0.011862 | 0.458793 | DOWN |
| SCNN1A | -1.02104 | 0.012042 | 0.460785 | DOWN |
| AKR1B10 | 1.712218 | 0.01206 | 0.460785 | UP |
| B3GALNT1 | -1.30593 | 0.012293 | 0.466042 | DOWN |
| COL8A1 | 1.605484 | 0.012375 | 0.466042 | UP |
| SLPI | -1.669 | 0.012626 | 0.468141 | DOWN |
| MAGEA1 | 1.806625 | 0.012722 | 0.468141 | UP |
| INHBB | -1.76499 | 0.01285 | 0.468141 | DOWN |
| ANXA3 | -1.92566 | 0.01286 | 0.468141 | DOWN |
| TMEM140 | -1.18086 | 0.012927 | 0.468141 | DOWN |
| HLA-DMB | -1.26654 | 0.013134 | 0.470081 | DOWN |
| HEY1 | 1.66977 | 0.01317 | 0.470081 | UP |
| MAGEA12 | 1.224906 | 0.013347 | 0.470081 | UP |
| KRT16 | -1.27614 | 0.013446 | 0.470081 | DOWN |
| SDPR | 1.627461 | 0.01357 | 0.470081 | UP |
| TLR3 | -1.12959 | 0.013598 | 0.470081 | DOWN |
| PRRX2 | 1.174506 | 0.013716 | 0.470124 | UP |
| TBR1 | 1.039672 | 0.013778 | 0.470124 | UP |
| IFITM1 | -1.44386 | 0.013907 | 0.470462 | DOWN |
| CST6 | -1.47098 | 0.014238 | 0.476616 | DOWN |
| RIMS1 | 1.111916 | 0.014312 | 0.476616 | UP |
| APBA2 | 1.040788 | 0.014341 | 0.476616 | UP |
| SFTPD | -1.03974 | 0.014359 | 0.476616 | DOWN |
| ANKRD7 | 1.274987 | 0.014449 | 0.476616 | UP |
| EIF1AY | 1.670883 | 0.014618 | 0.477486 | UP |
| FAM198B | 1.38643 | 0.01466 | 0.477486 | UP |
| OCLN | -1.07323 | 0.01474 | 0.477486 | DOWN |
| GJB5 | -1.04211 | 0.014859 | 0.477486 | DOWN |
| CRLF1 | 1.871457 | 0.014884 | 0.477486 | UP |
| CLGN | -1.23606 | 0.015065 | 0.480693 | DOWN |
| RHOD | -1.13567 | 0.015186 | 0.481927 | DOWN |
| FAIM3 | -1.05875 | 0.015648 | 0.487435 | DOWN |
| KLHL4 | 1.008668 | 0.015875 | 0.488183 | UP |
| INSM1 | 1.100386 | 0.016005 | 0.488183 | UP |
| ELMO1 | 1.041235 | 0.016138 | 0.488183 | UP |
| SH2B3 | 1.179835 | 0.016153 | 0.488183 | UP |
| ZEB2 | 1.051558 | 0.016184 | 0.488183 | UP |
| CYR61 | -1.05939 | 0.016201 | 0.488183 | DOWN |
| C4BPB | -1.4109 | 0.016228 | 0.488183 | DOWN |
| EPB41L3 | 1.467707 | 0.016292 | 0.488183 | UP |
| CDKN2B | -1.09097 | 0.016369 | 0.48823 | DOWN |
| ADAM23 | 1.002962 | 0.016562 | 0.488822 | UP |
| PSG7 | -1.0178 | 0.016652 | 0.490074 | DOWN |
| SERPINE2 | 1.29329 | 0.016749 | 0.490074 | UP |
| THSD7A | 1.383904 | 0.016884 | 0.490074 | UP |
| VEGFC | 1.119206 | 0.01709 | 0.490074 | UP |
| ITGA3 | -1.24596 | 0.017141 | 0.490074 | DOWN |
| ADH1C | -1.29495 | 0.017267 | 0.490074 | DOWN |
| HLA-DPB1 | -1.0953 | 0.017344 | 0.490074 | DOWN |
| OLFM1 | 1.266949 | 0.017411 | 0.490074 | UP |
| SCRN1 | -1.81652 | 0.017474 | 0.490074 | DOWN |
| DNMT3L | -1.02418 | 0.017491 | 0.490074 | DOWN |
| ALDH1A3 | -1.27717 | 0.017515 | 0.490074 | DOWN |
| ALDH3A1 | 1.533004 | 0.017516 | 0.490074 | UP |
| PPEF1 | 1.850244 | 0.01778 | 0.490458 | UP |
| GALNT6 | -1.15785 | 0.017847 | 0.490674 | DOWN |
| SGCE | -1.50634 | 0.018044 | 0.493124 | DOWN |
| UPK1A | 1.048125 | 0.018183 | 0.495776 | UP |
| LAMB1 | 1.03227 | 0.018288 | 0.497476 | UP |
| PPP1R14D | -1.21315 | 0.018369 | 0.498527 | DOWN |
| MCAM | 1.499204 | 0.018441 | 0.499335 | UP |
| CFI | -1.29712 | 0.01897 | 0.505559 | DOWN |
| MAOA | 1.285719 | 0.019071 | 0.506253 | UP |
| HYAL1 | 1.282448 | 0.019593 | 0.516318 | UP |
| ARMCX1 | -1.23372 | 0.019643 | 0.516501 | DOWN |
| C3 | -1.91629 | 0.019698 | 0.516777 | DOWN |
| PLA1A | -1.43579 | 0.020092 | 0.523628 | DOWN |
| ZNF165 | -1.07524 | 0.020325 | 0.526823 | DOWN |
| CYP4F11 | 1.286813 | 0.020764 | 0.526823 | UP |
| PPM1H | -1.00118 | 0.020971 | 0.526823 | DOWN |
| PDGFRB | 1.066094 | 0.021026 | 0.526823 | UP |
| TTC9 | -1.09277 | 0.021176 | 0.526823 | DOWN |
| UCP2 | -1.21802 | 0.021264 | 0.526823 | DOWN |
| IFI27 | -1.97613 | 0.021348 | 0.526823 | DOWN |
| ARNT2 | -1.14188 | 0.021375 | 0.526823 | DOWN |
| GPR110 | -1.1691 | 0.021519 | 0.52929 | DOWN |
| SCN3A | 1.042896 | 0.021638 | 0.529353 | UP |
| AADAC | 1.977953 | 0.02191 | 0.529353 | UP |
| IDO1 | -1.12956 | 0.022278 | 0.530676 | DOWN |
| ZNF468 | -1.34187 | 0.022385 | 0.530676 | DOWN |
| FOXF1 | 1.97308 | 0.02243 | 0.530688 | UP |
| PDGFRL | 1.083415 | 0.022692 | 0.532668 | UP |
| ALDH1A1 | 2.056725 | 0.02279 | 0.532668 | UP |
| IL18R1 | 1.496237 | 0.022838 | 0.532668 | UP |
| PSMB9 | -1.13606 | 0.023137 | 0.534539 | DOWN |
| MT1E | -1.18263 | 0.02339 | 0.53564 | DOWN |
| TGM2 | -1.1695 | 0.023392 | 0.53564 | DOWN |
| INSL6 | 1.027973 | 0.023521 | 0.53564 | UP |
| PDGFA | 1.114564 | 0.023568 | 0.53564 | UP |
| CLDN3 | -1.21398 | 0.023627 | 0.53564 | DOWN |
| TSPAN1 | -1.63045 | 0.023785 | 0.53564 | DOWN |
| LYPD1 | 1.230254 | 0.023954 | 0.53564 | UP |
| F3 | -1.14214 | 0.024331 | 0.53564 | DOWN |
| CFHR4 | 1.120211 | 0.024491 | 0.53564 | UP |
| SLC16A4 | 1.297765 | 0.025305 | 0.540361 | UP |
| TAPBPL | -1.04134 | 0.02573 | 0.540361 | DOWN |
| HGF | 1.394451 | 0.026258 | 0.543353 | UP |
| HOOK1 | -1.28359 | 0.026346 | 0.543353 | DOWN |
| PROM1 | -1.17017 | 0.026509 | 0.543353 | DOWN |
| TNNC1 | -1.66468 | 0.026775 | 0.545664 | DOWN |
| MX2 | -1.182 | 0.027747 | 0.553415 | DOWN |
| RAB3B | 1.055485 | 0.028067 | 0.55343 | UP |
| LEPREL2 | 1.198462 | 0.028182 | 0.55343 | UP |
| EFEMP1 | 1.001364 | 0.02851 | 0.55343 | UP |
| TNFSF10 | -1.37032 | 0.028545 | 0.55343 | DOWN |
| CDH13 | 1.60673 | 0.029173 | 0.556909 | UP |
| SLC19A3 | 1.078185 | 0.029584 | 0.562278 | UP |
| DPP4 | -1.24232 | 0.029659 | 0.562278 | DOWN |
| PRDM13 | 1.107003 | 0.029731 | 0.562278 | UP |
| OLFML1 | -1.01839 | 0.029814 | 0.562278 | DOWN |
| DNALI1 | -1.01105 | 0.030009 | 0.562278 | DOWN |
| TPPP3 | 1.209478 | 0.030208 | 0.56251 | UP |
| KDM5D | 1.560657 | 0.030303 | 0.56251 | UP |
| IL17RB | -1.29328 | 0.030677 | 0.56251 | DOWN |
| SLC6A15 | 1.325308 | 0.030708 | 0.56251 | UP |
| CNTN1 | 1.090381 | 0.030722 | 0.56251 | UP |
| EPCAM | -2.22516 | 0.031463 | 0.565353 | DOWN |
| PAPSS2 | 1.019932 | 0.031464 | 0.565353 | UP |
| HJURP | 1.124904 | 0.031538 | 0.565353 | UP |
| SYNC | -1.07447 | 0.031641 | 0.565353 | DOWN |
| FABP3 | -1.01515 | 0.032337 | 0.5688 | DOWN |
| FN1 | 1.17642 | 0.034467 | 0.583678 | UP |
| HCLS1 | 1.569022 | 0.034524 | 0.583678 | UP |
| LGSN | 1.401645 | 0.034926 | 0.586538 | UP |
| SLC6A14 | -1.2084 | 0.035327 | 0.590754 | DOWN |
| BDKRB1 | -1.23364 | 0.035542 | 0.592665 | DOWN |
| COCH | 1.197624 | 0.035806 | 0.593121 | UP |
| TUBAL3 | 1.424427 | 0.036177 | 0.595682 | UP |
| MX1 | -1.40562 | 0.036986 | 0.601457 | DOWN |
| UBE2L6 | -1.02945 | 0.037381 | 0.602108 | DOWN |
| FAS | -1.08859 | 0.037797 | 0.603831 | DOWN |
| MYO5C | -1.10433 | 0.037913 | 0.604532 | DOWN |
| CD226 | 1.117178 | 0.038062 | 0.604793 | UP |
| IL18RAP | 1.088528 | 0.038415 | 0.605424 | UP |
| PI3 | -1.79641 | 0.03891 | 0.608434 | DOWN |
| SNCA | 1.361841 | 0.03912 | 0.610514 | UP |
| MN1 | 1.718937 | 0.039267 | 0.610514 | UP |
| FST | 1.812754 | 0.040006 | 0.611795 | UP |
| CCL2 | -1.37345 | 0.040967 | 0.612676 | DOWN |
| SLC15A3 | -1.3135 | 0.041595 | 0.612676 | DOWN |
| ITM2A | -1.08827 | 0.043081 | 0.616836 | DOWN |
| MUC16 | -1.37075 | 0.043876 | 0.623186 | DOWN |
| CD70 | 1.192819 | 0.043918 | 0.623186 | UP |
| ICAM1 | -1.03852 | 0.044159 | 0.623934 | DOWN |
| ALPP | -1.53478 | 0.04436 | 0.623934 | DOWN |
| IFI6 | -1.21057 | 0.044552 | 0.623934 | DOWN |
| STC1 | 1.213363 | 0.04508 | 0.626141 | UP |
| PTX3 | -1.5277 | 0.045472 | 0.626604 | DOWN |
| CH25H | -1.04995 | 0.046537 | 0.627131 | DOWN |
| KLF5 | -1.10989 | 0.047807 | 0.629034 | DOWN |
| CSGALNACT1 | 1.214346 | 0.048327 | 0.629034 | UP |
| HLA-DPA1 | -1.18691 | 0.048556 | 0.629034 | DOWN |
| PTGDS | -1.13954 | 0.048558 | 0.629034 | DOWN |
| CXCL3 | 1.26577 | 0.049423 | 0.631735 | UP |
